# Fufang Muji Granules Ameliorate Liver Fibrosis by Reducing Oxidative Stress and Inflammation, Inhibiting Apoptosis, and Modulating Overall Metabolism

**DOI:** 10.3390/metabo14080446

**Published:** 2024-08-11

**Authors:** Lei Men, Zhihong Gu, Enhua Wang, Jiwen Li, Zhongyu Li, Keke Li, Chunbin Li, Xiaojie Gong

**Affiliations:** 1 Department of Biological Engineering, College of Life Science, Dalian Minzu University, Dalian 116600, China; menlei@dlnu.edu.cn (L.M.); 17864226819@163.com (Z.G.); wang_enhua@163.com (E.W.); ljw131009@163.com (J.L.); lzy@dlnu.edu.cn (Z.L.); likeke@dlnu.edu.cn (K.L.); lcb@dlnu.edu.cn (C.L.); 2 Key Laboratory of Biotechnology and Bioresources Utilization, Ministry of Education, Dalian Minzu University, Dalian 116600, China

**Keywords:** Fufang Muji granules, liver fibrosis, Manchu, metabolomics, bile metabolism

## Abstract

Fufang Muji granules (FMGs) are a prominent modern prescription Chinese patent formulation derived from the Muji decoction. Utilized in clinical practice for nearly four decades, FMGs have demonstrated efficacy in treating liver diseases. However, the precise mechanism of action remains unclear. This study investigates the hepatoprotective effects of FMGs against liver fibrosis in rats based on untargeted metabolomics and elucidates their underlying mechanisms. A comprehensive model of liver fibrosis was established with 30% CCl_4_ (2 mL/kg) injected intraperitoneally, and a fat and sugar diet combined with high temperatures and humidity. Rats were orally administered FMGs (3.12 g/kg/d) once daily for six weeks. FMG administration resulted in improved liver fibrosis and attenuated hepatic oxidative stress and apoptosis. Furthermore, FMGs inhibited hepatic stellate cell activation and modulated transforming growth factor β1/Smad signaling. Additionally, FMG treatment influenced the expression levels of interleukin-6, interleukin-1β, and tumour necrosis factor alpha in the injured liver. Metabolic pathways involving taurine and hypotaurine metabolism, as well as primary bile acid biosynthesis, were identified as mechanisms of action for FMGs. Immunohistochemistry, quantitative reverse transcription polymerase chain reaction (RT-qPCR), and quantitative analysis also revealed that FMGs regulated taurine and hypotaurine metabolism and bile acid metabolism. These findings provide a valuable understanding of the role of FMGs in liver fibrosis management.

## 1. Introduction

Liver fibrosis, a common pathological process in various liver diseases, arises from an imbalance in chronic injury healing and may progress to cirrhosis, liver failure, and portal hypertension. It often requires liver transplantation. The Global Burden of Disease Survey for 2023 highlighted cirrhosis as a significant contributor to global mortality, accounting for 1.82% of all deaths and causing 1.2 million fatalities annually [1]. Given the limited treatment options for advanced cirrhosis, early intervention in hepatitis and liver fibrosis development has become a clinical priority. In addition, the multifactorial nature and complex progression of liver fibrosis pose significant challenges in clinical management, necessitating a comprehensive understanding of its aetiology and exploration into therapeutic interventions [2,3,4,5,6]. There is currently no effective diagnostic or therapeutic strategy available. The current therapeutic approaches are predominantly focused on a single process or pathway of disease progression, with a limited impact on its reversal [2,3,4,5,6]. Traditional Chinese medicine’s complex nature, characterized by multiple targets and components, can precisely address the deficiencies of the current treatment of liver fibrosis.

Fufang Muji granules (FMGs), a well-known contemporary product rooted in Manchu ethnopharmacy and derived from the popular Muji decoction, have been clinically employed for nearly four decades in treating various liver ailments, including cirrhosis, hepatitis, and hepatocellular carcinoma. It is now included in Chinese Medicine Prescription Preparation, Vol. XVI [7,8]. Comprising a blend of four traditional Chinese medicinal herbs, namely *Coriolus versicolor* extract (known as Yunzhi in Chinese and as Muji in Manchu language), *Sophora tonkinensis*, *Cuscuta chinensis*, and *Juglans mandshurica* bark, FMGs’ pharmacological activity and mechanism remain understudied, despite extensive research on the individual herb’s pharmacological effects [9,10,11,12,13,14]. Clinical investigations have highlighted FMGs’ efficacy in enhancing hepatocyte defence, combating liver fibrosis, and exhibiting notable therapeutic effects [15]. Moreover, FMGs have been shown to modulate the transforming growth factor-β1(TGF-β1)/Smad pathway and influence immune and apoptotic cytokines to suppress hepatocellular carcinoma in rats [7]. However, further investigation into FMGs’ mechanism of action in ameliorating liver fibrosis or liver injury is lacking.

Chinese medicine posits that the pathogenesis of liver disease is primarily influenced by pathological factors such as dampness, heat, and epidemiological toxins. It is challenging to reproduce the multifaceted and complex syndromes observed in liver disease using single-factor-induced injury models. The mixed-factor-induced rat liver injury model offers a more comprehensive understanding of the mechanism of action of traditional Chinese medicine (TCM). Metabolomics emerges as a powerful tool for understanding traditional Chinese medicine’s pharmacological mechanisms. By modulating the activities and functions of organelles, organs, and organisms, metabolites and related metabolic pathways interact synergistically, illuminating the underlying molecular mechanisms of pharmacological action [16,17].

Oxidative stress is speculated to be a crucial pathological process responsible for the emergence and progression of various liver disorders [4]. According to reports, all four pharmaceutical components that make up FMGs have antioxidant properties [9,10,18,19,20,21]. Taurine and hypotaurine could prevent the formation of reactive oxygen species (ROS) and lipid peroxidation or defend the process of intracellular glutathione (GSH) storage replenishment during oxidative disorder, indirectly displaying antioxidant effects [22,23]. Taurine also plays a key role in maintaining bile homeostasis [24]. The disturbances caused by liver fibrosis in the biosynthesis of primary bile acids can lead to the accumulation of bile acids, glycocholic acid, and bile salts. This accumulation ultimately results in hepatocellular apoptosis and necrosis, exacerbating hepatic fibrosis [16,25]. In this study, we aim to evaluate FMGs’ hepatoprotective effects on a comprehensive model of liver fibrosis in rats and to explore the underlying mechanisms through untargeted metabolomics analysis. The levels of taurine and bile acids were also assessed to elucidate the hepatoprotective mechanisms. Furthermore, the expression levels of liver fibrosis-related genes and indexes have been employed to elucidate hepatoprotective mechanisms.

## 2. Materials and Methods

### 2.1. Chemical Compounds and Reagents

FMGs (Lot No. 201101, Liaoning Dandong Pharmaceutical Group, Dandong, China) were purchased from Dalian Yichuntang Pharmacy. Commercial biochemical assay kits were purchased from the Nanjing Jiancheng Bioengineering Institute (Nanjing, China) and Elabscience Biotechnology Co., Ltd. (Wuhan, China). Olive oil was sourced from Shanghai Kerry Food Industry Co., Ltd. (Shanghai, China), and colchicine (Lot No. RH530750) and carbon tetrachloride (Lot No. C12709759) were sourced from Shanghai McLean Biochemical Technology Co. (Shanghai, China). The bile acids used in this article were obtained from Shanghai Yuanye Bio-Technology Co., Ltd. (Shanghai, China) or J&K Scientific. Additionally, reagents, including the TUNEL (terminal transferase mediated notch-end labelling) cell apoptosis detection kit, TGF-β1, bile salt export pump (Bsep), α-smooth muscle actin (α-SMA), antibody, and IgG-HRP multimer antibody, were purchased from Wuhan ServiceBio Technology Co., Ltd. (Wuhan, China). Acetonitrile, methanol, acetic acid, and formic acid were acquired from Merck Co. Inc. (Darmstadt, Germany). Distilled water was provided by Guangzhou Watsons Company (Guangzhou, China). All other reagents were of analytical grade.

### 2.2. Determination of Chemical Composition and Content

Thirty-six chemical constituents in FMGs were identified using liquid chromatography coupled with linear quadrupole ion trap-orbitrap mass spectrometry (LC-LTQ-Orbitrap/MS) [8]. The contents of several key components in FMGs were quantified using HPLC methods, as described in our previous studies [26,27,28].

### 2.3. Animal Study

**Figure 1:**
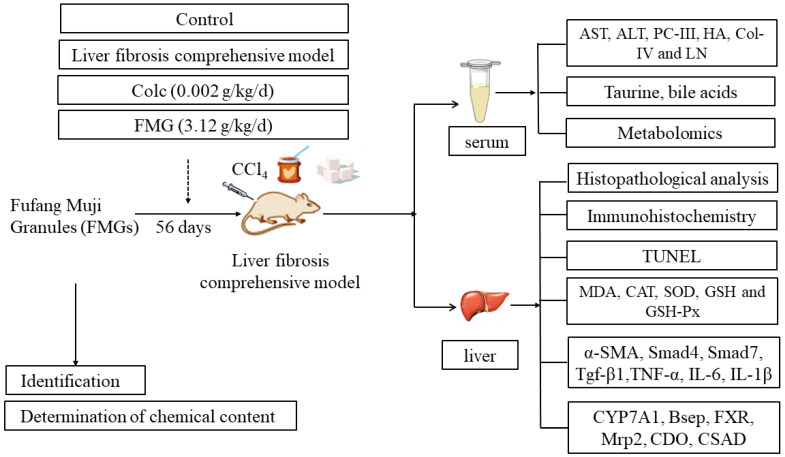
Experimental flow chart.

The Institutional Animal Care and Use Committee of Dalian Minzu University gave its approval for the animal study on 10 March 2021 (approval code 2020-0001). Male Sprague-Dawley (SD) rats (180–220 g) were provided by Liaoning Changsheng Biotechnology Co., Ltd. (Shenyang, China). Twenty-four rats were randomly assigned to four groups: control, colchicine, model, and FMG, with six rats per group. The control group was maintained on a standard diet at 25 ± 2 °C with a relative humidity of 50 ± 10%. Rats in the other three groups were fed a high-fat, high-sugar diet combined with a normal diet (1:1) at 28 ± 2 °C with a relative humidity of 90 ± 5%. After 15 days, the control group received intraperitoneal injections of olive oil, while the colchicine, model, and FMG groups received intraperitoneal injections of 30% CCl_4_ in olive oil suspension (2 mL/kg) twice weekly [29,30,31]. The colchicine and FMG groups were administered colchicine (0.002 g/kg/d) and FMG aqueous solution (3.12 g/kg/d, based on the clinical dose), respectively, via gavage daily for the following six weeks. The control and model groups received daily saline administration via gavage. Throughout the experiments, the rats’ mental condition, behavioural activity, coat colour, diet, and weight were monitored each morning. Rats were euthanised by intravenous injections of urethane, and blood and serum samples were promptly collected. The remaining liver tissues were stored at −80°, with a small portion preserved in a 4% paraformaldehyde solution for further examination. The experimental flowchart is shown in Figure 1.

### 2.4. Histopathological Analysis

Histopathologic alterations in the liver were examined using haematoxylin and eosin (HE) staining. Liver tissues were fixed in paraformaldehyde overnight, embedded, dehydrated, sectioned, and stained. Each rat’s liver tissue was observed under an optical microscope to identify histopathologic changes.

### 2.5. Serum and Liver Biochemistry Analysis

Serum levels of alanine aminotransferase (ALT) and aspartate aminotransferase were quantified as liver function parameters using biochemical kits from the Nanjing Jiancheng Bioengineering Institute (Nanjing, China). Serum levels of laminin (LN), type IV collagen (Col-IV), type III procollagen (PC-III), and hyaluronic acid (HA) were measured as liver fibrosis markers using ELISA kits (Elabscience Biotechnology Co., Ltd., Wuhan, China). A taurine ELISA kit was purchased from ELK Biotechnology (Wuhan, China). Hepatic levels of malondialdehyde (MDA), superoxide dismutase (SOD), glutathione (GSH), glutathione peroxidases (GSH-Px), and catalase (CAT) were determined as oxidative parameters according to the manufacturer’s instructions (Nanjing Jiancheng Bioengineering Institute, Nanjing, China).

### 2.6. Immunohistochemistry Analysis

Liver sections were deparaffinised, and endogenous peroxidase was blocked using 3% H_2_O_2_ (Shandong Anjie Gaoke Sterilization Technology Co., Dezhou, China). Tissue antigen was retrieved using a microwave with a 0.01 mol/L citric acid antigen retrieval solution. After cooling to room temperature, primary antibodies (α-SMA, TGF-β1, and Bsep) were added and incubated overnight. The sections were then incubated with the secondary antibody (IgG-HRP polymer antibody) for 1–2 h at a warm temperature, followed by washing with phosphate buffered saline. Colour development was performed using 3,3′-diaminobenzidine (DAB), and the samples were sealed with neutral gum before observation under a light microscope.

### 2.7. TUNEL Analysis

Hepatocyte apoptosis was identified using deoxyribonucleotide-terminal transferase-mediated notch-end labelling (TUNEL). All steps were performed following the kit’s instructions. Deparaffinised and rehydrated rat liver tissue sections were treated with proteinase K, TUNEL reaction solution, 4′,6-diamidino-2-phenylindole (DAPI) for nuclei staining, and haematoxylin for counterstaining. Images were then captured using a fluorescence microscope.

### 2.8. HPLC-MS/MS-Based Metabolomics

#### 2.8.1. Sample Preparation

A total of 400 μL of an acetonitrile:methanol (1:1) solution was added to a centrifuge tube containing 100 μL of serum. The mixture was vortexed for 30 s, followed by ultrasound extraction (5 °C, 40 KHz) for 30 min. The sample was then cooled at −20 °C for 30 min, centrifuged at 4 °C and 13,000× *g* for 15 min, and the supernatant was dried under a nitrogen stream. The residue was redissolved in 100 μL of a 1:1 mixture of acetonitrile and water and extracted using low-intensity ultrasound for 5 min (5 °C, 40 KHz). After a final centrifugation at 4 °C and 13,000× *g* for 5 min, the supernatant was analysed using LC-MS/MS.

#### 2.8.2. HPLC-MS/MS Method

Serum samples were analysed using an ultra-performance liquid chromatography (UPLC) system (Waters, USA) on an HSS T3 column (2.1 mm × 100 mm, 1.8 μm) at 40 °C. The mobile phase composition and HPLC elution conditions are detailed in Table S1. The injection volume was 2 μL.

Mass spectrometry was performed utilising a UPLC-Q Exactive HF-X mass spectrometer with both positive and negative ion scanning modes, covering a scan range of 70 to 1000 *m*/*z*. Sheath gas and auxiliary gas flow rates were set at 48 psi and 14 psi, respectively. The auxiliary gas heating temperature was 430 °C, the ion spray voltage was ±3500 V, the ion transport tube temperature was 325 °C, and the normalised collision energy was 20–40–60 V cyclic collision energy.

#### 2.8.3. Data Analysis

The intensities of individual ion fragments were normalised to the total peak intensity in the relative chromatogram. Variables with a relative standard deviation (RSD) of less than 30% in the QC samples were retained for multivariate statistical analysis. Raw data were processed in Progenesis QI (Waters Corporation, Milford, MA, USA) for baseline filtering, peak identification, integration, retention time correction, and peak alignment. This processing resulted in a data matrix containing retention time, mass-to-charge ratio, and peak intensity.

A Student’s *t*-test and multiplicative analysis of variance were performed. Differential metabolites were identified based on variable importance in projection (VIP) values obtained from the OPLS-DA model and Student’s *t*-test *p*-value, with metabolites meeting VIP > 1 and *p* < 0.05 considered significant.

Metabolite information was obtained by matching MS and MS/MS data against the public metabolic databases HMDB (http://www.hmdb.ca/ (accessed on 3 January 2022), Metlin (https://metlin.scripps.edu/ (accessed on 3 January 2022), and the Meggie custom database. The processed data matrix was uploaded to the Meggie Bio Cloud platform (https://cloud.majorbio.com (accessed on 10 January 2022) for further analysis. Principal component analysis (PCA), partial least squares discriminant dnalysis (PLS-DA) and orthogonal least squares discriminant analysis (OPLS-DA) were performed using the R package (Version 1.6.2). Additionally, a Student’s *t*-test and multiplicative analysis of variance were performed. Differential metabolites were identified based on variable importance in projection (VIP) values obtained from the OPLS-DA model and Student’s *t*-test *p*-value, with metabolites meeting VIP > 1 and *p* < 0.05 considered significant.

Metabolic pathway annotation for differential metabolites was conducted using the KEGG database (https://www.kegg.jp/kegg/pathway.html (accessed on 18 January 2022). Pathway enrichment analysis was performed using the Python package scipy.stats (v1.14.0 Manual), and Fisher’s exact test was used to identify the most relevant pathways associated with the experimental treatments.

### 2.9. Quantitative Real-Time PCR (qPCR) Analysis

Total RNA was extracted using AG RNAex Pro reagent (Accurate Biotechnology Co., Ltd., Changsha, China). Following extraction, cDNA synthesis was performed using the Evo M-MLV RT Mix kit with gDNA Clean for qPCR Ver.2 from Accurate Biotechnology Co., Ltd., following the manufacturer’s instructions. Primers were designed using Primer Premier 5.0 software, with efficiencies ranging from 90% to 105%. qPCR was conducted on an Applied Biosystems 7500 Real-Time PCR System (Thermo Fisher Scientific, Shanghai, China) using the SYBR Green Premix Pro Taq HS qPCR kit (Accurate Biotechnology Co., Ltd., Changsha, China). Each sample was tested in triplicate, and three independent biological samples were analysed. The 2^−ΔΔCt^ method was used to quantify relative gene expression, which was normalised to β-actin expression. Gene primer sequences are provided in Table 1.

### 2.10. Determination of Bile Acid Levels in Serum

The procedure for extracting bile acids from serum samples was conducted in accordance with the HPLC-MS/MS-based metabolomic method. Bile acids were detected using an AcQuity UPLC I-class/QTRAP 6500 + system (AB SCIEX, Shanghai, China). The mobile phase consisted of acetonitrile and water (0.3% acetic acid). The optimized MRM parameters for the determination of bile acids are presented in Table 2.

### 2.11. Statistical Analysis

Results are reported as mean ± SD and analysed via one-way ANOVA (analysis of variance) in GraphPad Prism 9 (Boston, MA, USA) to determine *p*-values. The Dunnett *t*-test was used to compare the two groups. Statistical significance was set at *p* < 0.05 for significant differences, *p* < 0.01 for highly significant differences, and *p* < 0.001 for extremely significant differences. Statistical analyses were performed using GraphPad Prism 9 software (Boston, MA, USA).

## 3. Results

### 3.1. Ingredient Identification and Quality Control Results of FMGs

Totally thirty-six major chemical compounds, including twelve alkaloids, thirteen flavonoids, three naphthalene quinones, six phenolic acids, and two other compounds, were identified or inferred in FMGs (Figure S1 and Table S2). The results of the determination of the content of the key components are presented in Figures S2 and S3 and Tables S3.

### 3.2. FMGs Alleviate Hepatic Pathological and Functional Changes in Liver Fibrosis

**Figure 2:**
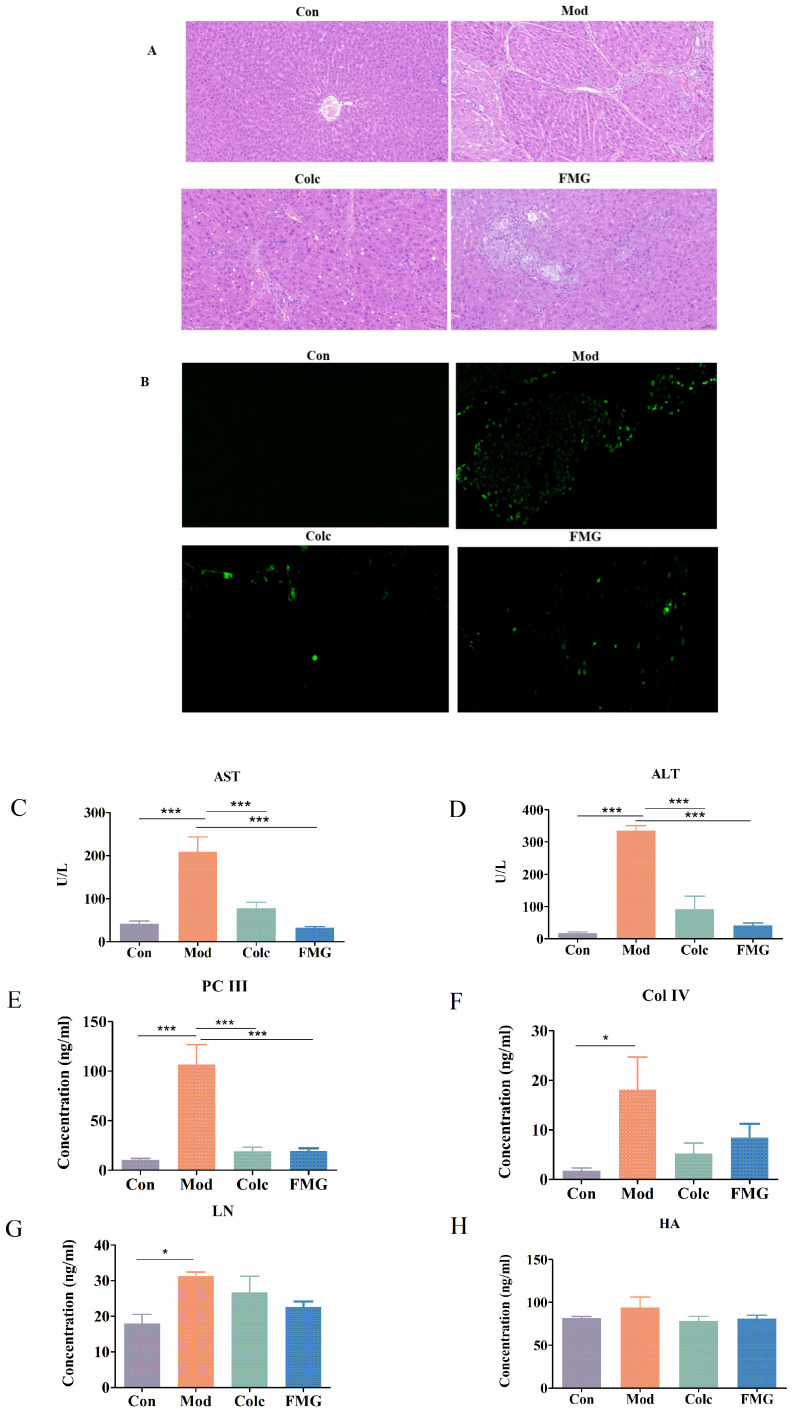
Protective effect of FMGs on liver fibrosis in rat livers. (**A**) Histopathologic analysis of livers (400× magnification). (**B**) Effects of FMGs on the apoptosis of rat hepatocytes (TUNEL staining, 400× magnification). (**C**,**D**) Liver function parameters assessed by (alanine transaminase, ALT) and (aspartate aminotransferase, AST). (**E**–**H**) Liver fibrosis markers assessed by laminin (LN), type IV collagen (Col-IV), type III procollagen (PC-III), and hyaluronic acid (HA). (n = 6, mean ± SD, * *p* < 0.05, *** *p* < 0.001; Con, control group; Mod, model group; Colc, colchicine group; FMG, FMG group).

HE staining (Figure 2A) revealed no significant abnormalities in the portal region between adjacent liver lobules and no inflammatory changes in the control group. Hepatocytes in the control group were rounded and full and arranged in a regular and orderly manner, with no obvious dilation or extrusion of the hepatic sinusoids. In contrast, rats in the model group exhibited cellular swelling, homogeneous, unstructured eosinophilic material in the cytoplasm, and extensive connective tissue hyperplasia around numerous confluent areas. Locally, these lesions were accompanied by small amounts of brownish-yellow pigmentation and visible foci of hepatocellular gross vitreous lesions. In the FMGs or colchicine-treated group, fibrogenesis and hepatic pathology were significantly reduced compared to the model group.

The TUNEL labelling procedure (Figure 2B) assessed hepatocyte apoptosis. After FMG treatment, the number of apoptotic-positive cells decreased and was closer to that of the control group, suggesting that FMGs reduce liver cell apoptosis, thereby mitigating liver damage.

Additionally, ALT and AST levels significantly increased in the model group. FMG treatment significantly inhibited the increase in these liver function indexes (*p* < 0.001, Figure 2C,D).

### 3.3. FMGs Ameliorate Liver Fibrosis in Rats

The levels of four liver fibrosis markers (PC-III, HA, Col-IV, and LN) were elevated to varying degrees in the model group. However, FMG treatment successfully suppressed the increase of these markers (Figure 2E–H), indicating that FMGs can ameliorate fibrotic liver injury.

### 3.4. FMGs Ameliorate Myofibroblast Activation in Liver Fibrotic Rats

**Figure 3:**
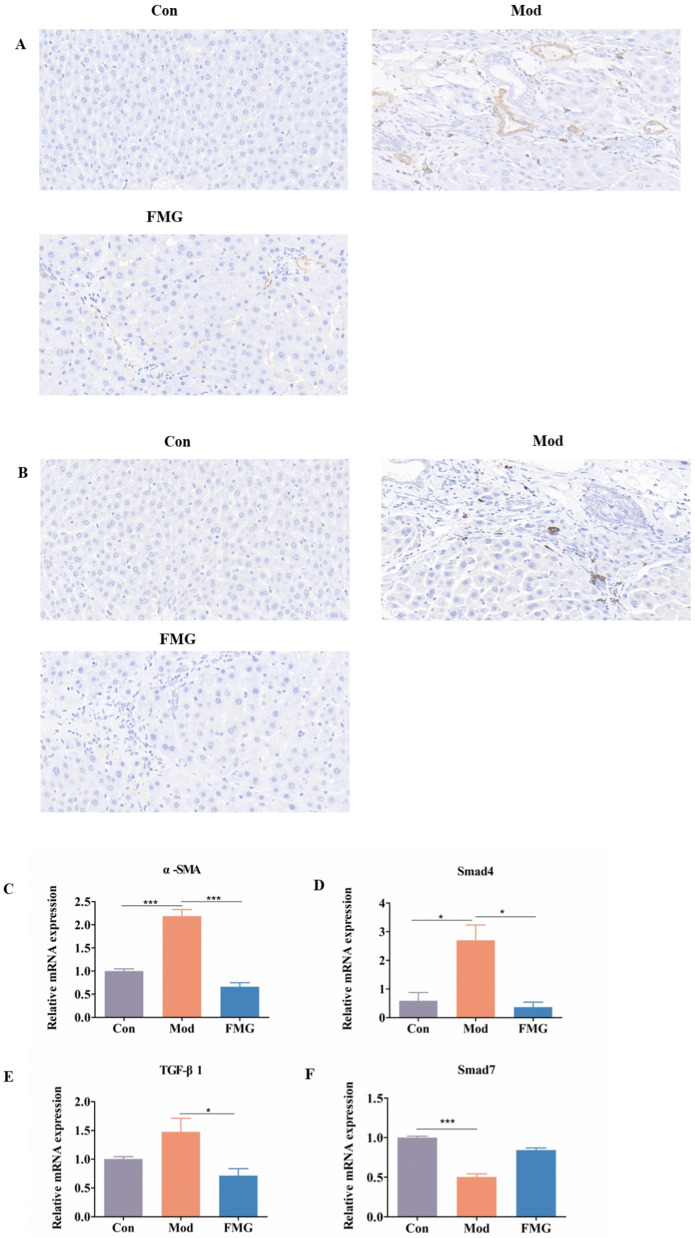
Immunohistochemical staining of α-smooth muscle actin (α-SMA) (**A**) and transforming growth factor β1 (TGF-β1) (**B**) (magnification 400×). Effects of FMG on the mRNA expression of α-SMA, TGF-β1, and SMAD family members 4 (Smad4) and 7 (Smad7) in the liver tissues measured using qPCR (**C**–**F**), n = 3, mean ± SD, * *p* < 0.05, *** *p* < 0.001; Con, control group; Mod, model group; FMG, FMG group.

Alpha-smooth muscle actin (α-SMA) serves as a marker protein for hepatic stellate cell activation, which is strongly associated with liver fibrosis. The TGF-β1/Smad pathway, a crucial signal transduction route in fibrosis, plays a significant role in regulating its progression. Immunohistochemical labelling revealed that the expression levels of TGF-β1 and α-SMA were significantly increased in the model group. FMG treatment resulted in significant reductions in the expression levels of both TGF-β1 and α-SMA (Figure 3A,B). Corresponding changes were observed in the hepatic mRNA expressions of α-SMA, TGF-β1, and Smad4 (*p* < 0.05 or 0.001 vs. model group, Figure 3C–E). Although the expression level of inhibitory Smad7 mRNA increased in FMG-treated rats, this change was not statistically significant (Figure 3F). These results suggest that FMGs’ alleviating effect is associated with the inhibition of hepatic TGF-β1/Smad signalling and myofibroblast activation.

### 3.5. FMGs Ameliorate Oxidative Stress Caused by Liver Fibrosis in Rats

**Figure 4:**
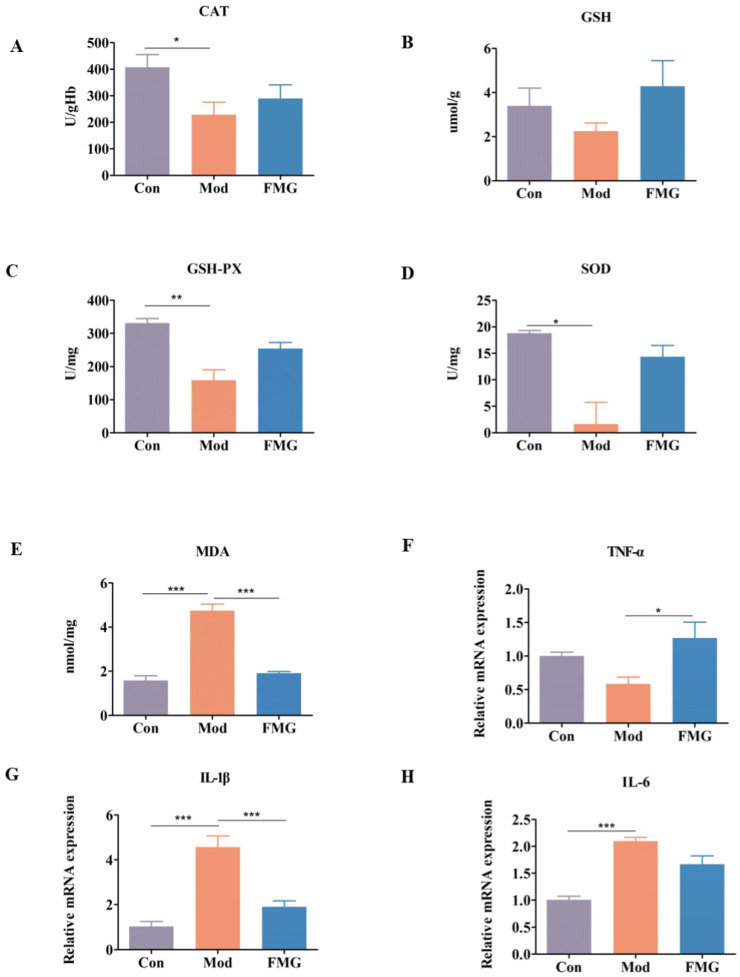
FMGs ameliorate oxidative stress and inflammatory factors in hepatic fibrosis rats. (**A**–**E**) The levels of catalase (CAT), glutathione (GSH), glutathione peroxidases (GSH-Px), superoxide dismutase (SOD), and malondialdehyde (MDA) in the liver were detected by detection kits. (**F**–**H**) Effects of FMGs on the mRNA expression of tumour necrosis factor α (TNF-α), interleukin 1β (IL-1β), and interleukin 6 (IL-6) in liver tissues measured using qPCR. (n = 3, mean ± SD, * *p* < 0.05, ** *p* < 0.01, *** *p* < 0.001; Con, control group; Mod, model group; FMG, FMG group).

The antioxidant capacity of FMGs was assessed in rats by measuring the activities of oxidative stress markers MDA, CAT, SOD, GSH, and GSH-Px in liver homogenate. Compared to the control group, liver fibrosis significantly increased the content or activity of SOD, GSH, CAT, and GSH-Px while significantly decreasing the activity of MDA (*p* < 0.001, Figure 4A–E). Following FMG treatment, all indices approached normal values, indicating FMGs’ ability to counteract oxidative stress by regulating antioxidant enzyme activity and lipid peroxidation in liver injury.

### 3.6. FMGs Reduce Inflammatory Factors in Hepatic Fibrotic Rats

The levels of interleukin 1β (IL-1β), interleukin 6 (IL-6), and tumour necrosis factor α (TNF-α) in the liver were quantified in the three rat groups using qPCR (Figure 4F–H). After suffering from liver fibrosis, the levels of IL-1β and IL-6 in the liver were significantly increased (*p* < 0.001 vs. control group). These levels were significantly reduced after FMG treatment. However, the trend of TNF-α expression level changes was the opposite.

### 3.7. FMGs Reverse the Metabolic Profiles of Hepatic Fibrotic Rats

**Figure 5:**
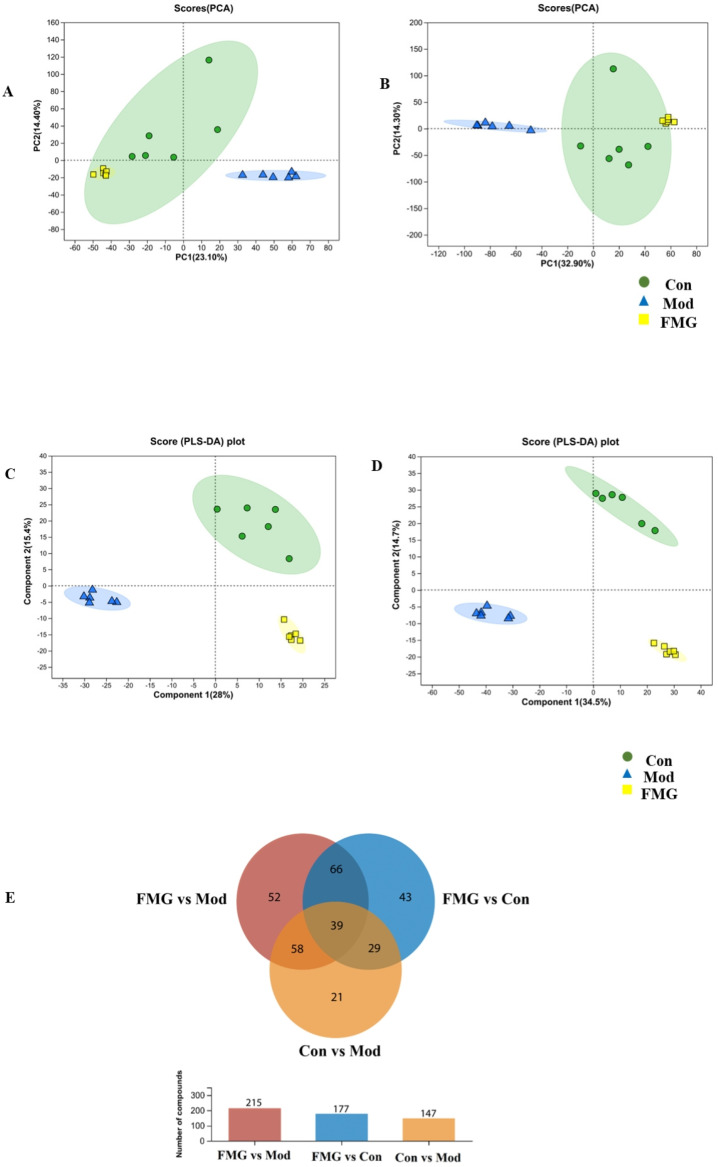
FMGs reverse the metabolic profiles of hepatic fibrosis rats. The PCA plots of the three groups in the positive (**A**) and negative (**B**) ion modes (PC1 represents the linear combination with the highest variance in the original data set; PC2 represents the linear combination with the highest variance in the direction orthogonal to the direction in which the first principal component is located.) The PLS-DA score plots of the three groups in the positive (**C**) and negative (**D**) ion modes (Component1represents the direction of the greatest variability in the data set; Component2 represents the direction of the second-greatest variability in the data). (**E**) A cross-comparison of three groups of differential metabolites was conducted using venn diagrams. (Con, control group; Mod, model group; FMG, FMG group).

Untargeted serum metabolomics analysis revealed that FMGs significantly altered the metabolic profiles of the three groups. PCA and OPLS-DA indicated clear differences between the control, model, and FMG groups. The PCA scores of the serum samples (Figure 5A,B) demonstrated a significant separation among the groups, especially between the control and model groups, with the FMG group showing a trend towards the control group.

The PLS-DA plots (Figure 5C,D) mirrored the trend observed in the PCA plots, displaying clear class discrimination with statistical parameter values of 0.955 and 0.846 for R2Y and Q2 in positive ion mode and 0.997 and 0.946 in negative ion mode (Figure S4). OPLS-DA (VIP > 1) combined with one-way ANOVA (*p* < 0.05) identified differential metabolites related to the hepatoprotective effect of FMGs on liver fibrosis. Thirty-nine metabolites with the greatest impact were identified through cross-comparison (Figure 5E). These metabolites included bile acids, alcohols and their derivatives, amino acids, fatty acid esters, and flavonoids. Among them, thirty-three metabolites showed increased abundance due to liver fibrosis but significantly decreased following FMG treatment (Figure S5 and Table S6). Conversely, the relative abundance of N-methylanthranilic acid, acetylbalchanolide, and DL-2-hydroxystearic acid showed an opposite trend, with FMG treatment reversing the downward trend caused by liver fibrosis. Additionally, the abundance of 1-arachidonoyl-2-hydroxy-sn-glycero-3-phosphate and oryzalexin E continued to decline post-FMG treatment, whereas the abundance of 25-hydroxyvitamin D3-26,23-lactone kept rising.

### 3.8. Metabolic Pathway Analysis

**Figure 6:**
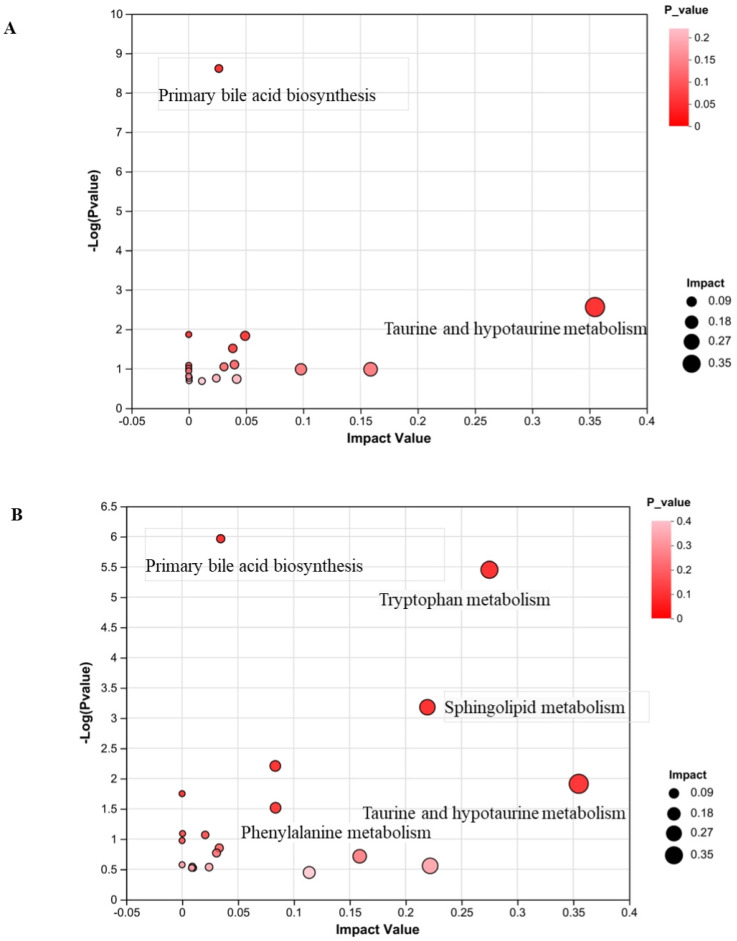

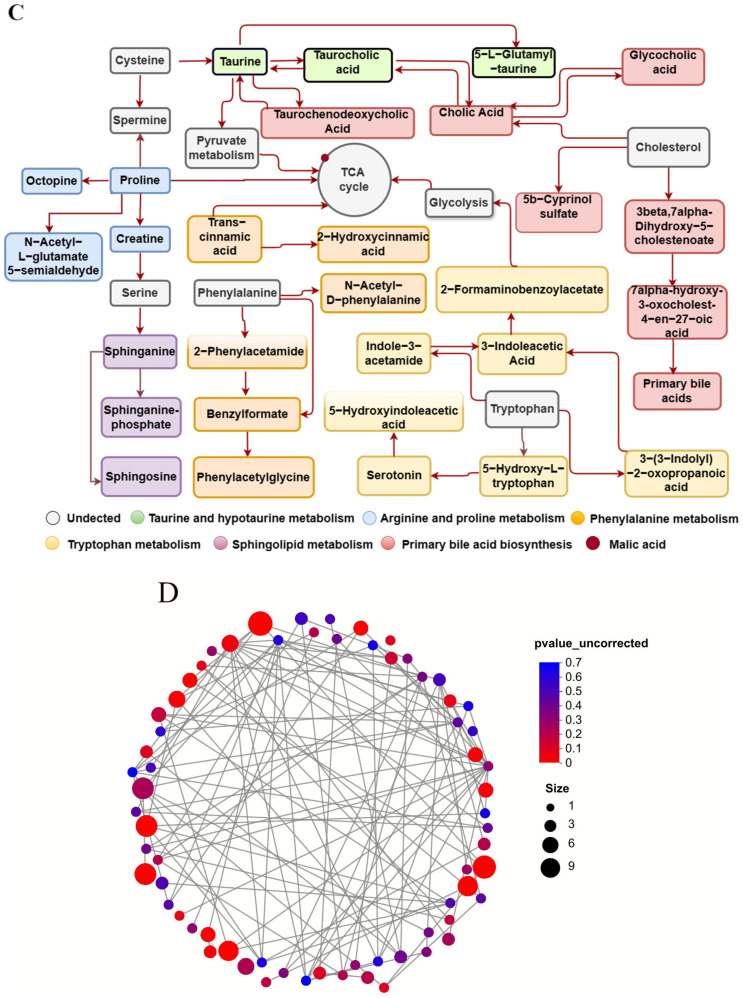
Metabolites pathway analysis in the liver. (**A**) Metabolites pathway impact between control and model groups. (**B**) Metabolites pathway impact between model and FMG group. (**C**) Schematic representation of the metabolic network of FMG regulation in rats based on the KEGG database. (**D**) Metabolic pathway network diagram.

Pathway analysis of differential metabolites revealed that liver disease and FMG treatment significantly impacted taurine and hypotaurine metabolism, as well as primary bile acid biosynthesis (*p* < 0.05) (Figure 6A,B). These results suggested that these pathways could be potential therapeutic targets. Figure 6C,D illustrates a multiple-correlation metabolic network study of liver fibrosis and FMG modulation.

### 3.9. FMGs Regulate Taurine and Hypotaurine Metabolism in Liver Fibrotic Rats

**Figure 7:**
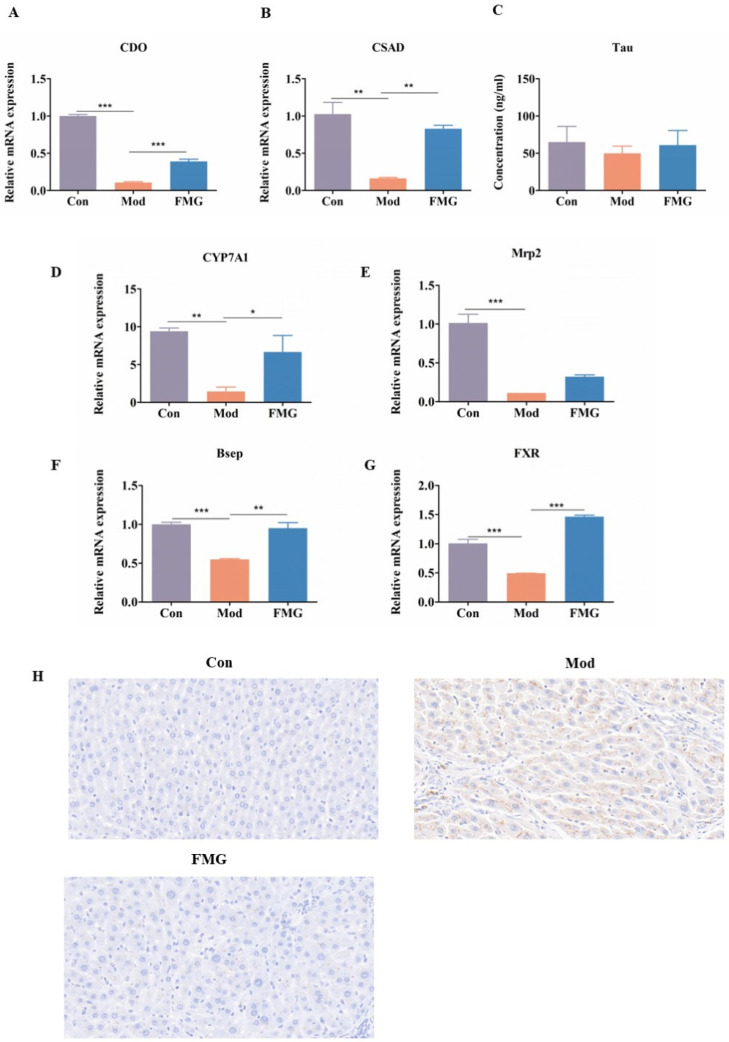
FMGs regulate taurine metabolism and bile metabolism. (**A**,**B**) Effects of FMGs on the mRNA expression of cysteine dioxygenase (CDO) and cysteinesulfinic acid decarboxylase (CSAD); (**C**) The level of taurine (Tau) in serum assessed by ELISA kits. (**D**–**G**) Effects of FMGs on the mRNA expression of bile salt export pump (Bsep), cholesterol 7 alpha-hydroxylase cytochrome p450 7A1 (CYP7A1), multidrug resistance protein 2 (Mrp2), and farsenoid X receptor (FXR) in the liver tissues measured using qPCR. (**H**) Immunohistochemical staining of Bsep (magnification 400×). (n = 3, mean ± SD, * *p* < 0.05, ** *p* < 0.01, *** *p* < 0.001; Con, control group; Mod, model group; FMG, FMG group).

Taurine synthesis in the body involves the enzymes cysteine dioxygenase (CDO) and cysteinesulfinic acid decarboxylase (CSAD). This study assessed the expression of CDO and CSAD in rat livers (Figure 7A,B). In the model group, liver expression levels of CDO and CSAD were significantly lower compared to the control group. FMG treatment significantly reversed these levels compared to the model group, suggesting FMGs’ role in regulating taurine metabolism by enhancing CDO and CSAD expression. This result was consistent with the ELISA quantification of taurine in serum (Figure 7C).

### 3.10. FMGs Regulate Bile Metabolism in Liver Fibrotic Rats

Key genes involved in primary bile acid biosynthesis and bile metabolism include bile salt export pump (Bsep), cholesterol 7 alpha-hydroxylase cytochrome p450 7A1 (CYP7A1), multidrug resistance protein 2 (Mrp2), and farsenoid X receptor (FXR). The expression of Bsep, CYP7A1, Mrp2, and FXR in the liver was determined using q-PCR. As shown in Figure 7D–G, liver fibrosis development significantly reduced the expression of Bsep, CYP7A1, Mrp2, and FXR (*p* < 0.001 vs. control group). FMG treatment reversed the expression levels of these genes to near-normal levels compared to the model group. However, immunohistochemical staining indicated an increase in Bsep expression due to liver fibrosis, while FMG administration resulted in a significant decrease in Bsep expression (Figure 7H).

Table 3 and Table 4 display the quantification of ten bile acids in serum. Among them, CA, GCA, GDCA, α-MCA, T-α-MCA, TCA, and TCDCA were significantly increased in the model group (*p* < 0.001, *p* < 0.05, or *p* < 0.01 vs. control group). CDCA and TUDCA exhibited an increasing trend in the model group compared to the control group, although there was no significant difference. However, DCA demonstrated an inverse trend. Following FMG treatment, the alterations in the concentrations of various bile acids were reversed.

## 4. Discussion

**Figure 8:**
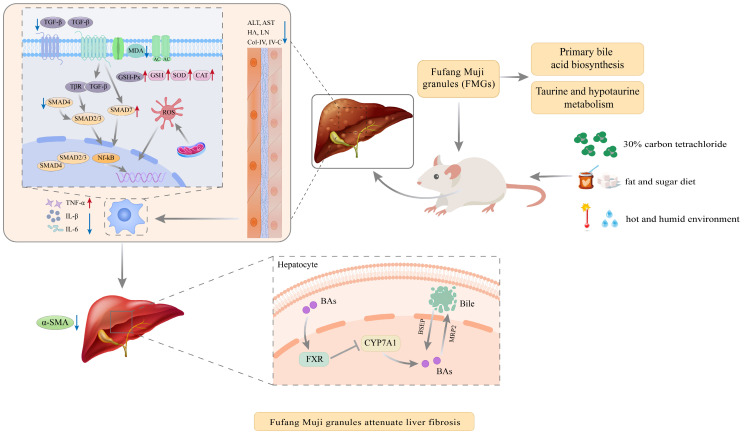
The possible mechanism of FMGs in the treatment of liver fibrosis.

**Figure 9:**
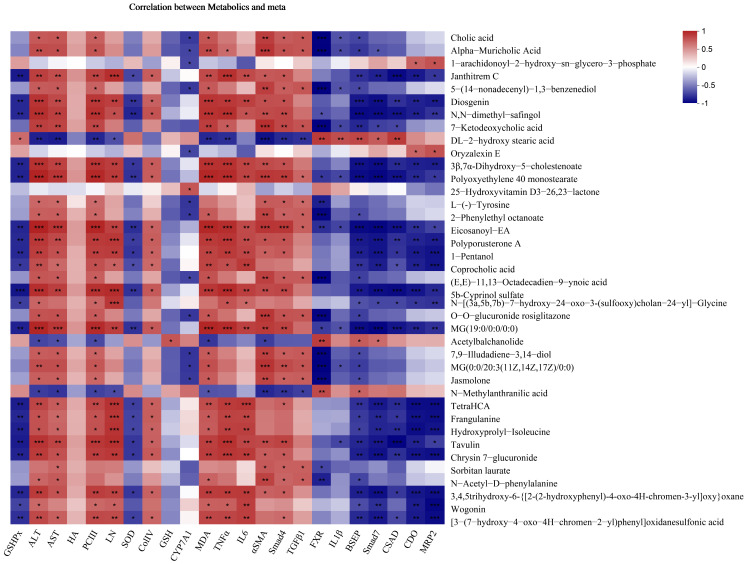
Pearson correlation analysis of biochemical parameters and potential biomarker metabolites. Blue squares indicate significant negative correlations, and red squares indicate significant positive correlations (*** *p* < 0.001, ** *p* < 0.01, * *p* < 0.05, correlation significance between two variables).

Liver fibrosis results from repeated or prolonged hepatic injury and is a wound-healing response that can progress to cirrhosis, liver cancer, or liver failure if left untreated. Preventing and treating liver fibrosis is crucial to avoiding these serious complications [5]. FMGs, a well-known herbal prescription with distinct Manchu characteristics, are used in China as a hepatoprotective medication for treating liver diseases [7]. This study revealed that FMGs attenuated comprehensive factors-induced hepatic fibrosis primarily by reducing hepatic inflammation, oxidative stress, and hepatic stellate cell (HSC) activation, affecting the TGF-1/Smads signalling pathway, controlling taurine and hypotaurine metabolism, and regulating bile metabolism (Figure 8). The correlation analysis further confirmed that the differential metabolites were strongly associated with liver fibrosis biochemical indicators (Figure 9).

The single-factor-induced liver fibrosis model presents significant difficulties in accurately representing the complex and multifaceted syndromes associated with the disease. Consequently, the present study sought to develop a comprehensive model of hepatic fibrosis by combining carbon tetrachloride, high-fat and high-sugar diets, and high temperatures and humidity. The model group displayed significantly elevated levels of Col-IV, HA, PC-III, and LN, as well as increased expression of α-SMA, a hepatic fibrosis marker, indicating the successful establishment of the liver injury and fibrosis model. FMG treatment brought these liver fibrosis indexes down to normal levels, demonstrating FMGs’ anti-fibrosis properties.

Oxidative stress is speculated to be a crucial pathological process responsible for the emergence and progression of various liver disorders [4]. When the liver is damaged, high-activity metabolism triggers the activation of free radicals and other substances, leading to the production of large amounts of ROS and increasing the production of MDA. This increase disrupts the balance between oxidants and reducing agents (antioxidants), reducing antioxidant capacity. Moreover, liver fibrosis can be treated by reversing this imbalance between oxidation and antioxidation, which is efficiently achieved by the sophisticated antioxidant defence mechanisms found in cells. These systems include enzymatic and non-enzymatic antioxidants. GSH works in tandem with enzyme-based antioxidants such as SOD, CAT, and GSH-Px to prevent damage from ROS or free radicals [32,33]. In this study, by modulating the activity of antioxidant enzymes and the levels of GSH, FMGs were found to significantly reduce oxidative damage. The four pharmaceutical components of FMGs possess antioxidant properties [9,10,18,19,20,21], indicating that FMGs exert a significant antioxidative effect in the treatment of liver fibrosis.

HSCs may become activated and undergo extensive proliferation in response to liver damage. The presence of α-SMA serves as an indicator of HSC activation. The primary initiator of α-SMA activation is the TGF-β/Smad signalling pathway [34,35]. The progression of liver disease, from early liver injury to inflammation and fibrosis to cirrhosis and cancer, is influenced by TGF-β signalling at every stage. TGF-β1 activates HSCs and promotes the production of extracellular matrix, leading to liver fibrosis [34,35]. Additionally, TGF-β1 can regulate fibroblasts into myofibroblasts and induce collagen synthesis by interacting with downstream cytoplasmic transcription factors, namely Smads [36,37]. Smad3 and Smad4 are fibrosis-promoting genes downstream of TGFβ1, with Smad4 increasing fibrosis development while Smad7 acts as a negative regulator, impeding the pro-fibrotic impacts of TGF-β [36]. The TGF-β1/Smad signalling pathway is considered the primary mechanism driving HSC activation and fibrogenesis. In this study, FMGs inhibited TGF-β1 and Smad4 expression while increasing Smad7 expression in the injured rat liver, demonstrating their efficacy in treating liver fibrosis.

During the development of hepatic fibrosis, a multitude of inflammatory signals are initiated, prompting the transformation of hepatic stellate cells into myofibroblasts. In this study, FMGs were able to reduce IL-6 and IL-1β levels in CCl_4_-induced rats, alleviating inflammatory responses. However, the levels of TNF-α exhibited an opposite trend in the liver. This dual role of TNF-α could be attributed to its pleiotropic nature, as it can initiate multiple signalling pathways involved in tissue inflammation, cell proliferation, and apoptosis. The concentration of TNF-α in the liver varies based on the aetiology of liver injury. Our findings suggest that TNF-α exerts a dual effect on liver injury. When TNF-α concentration reaches a certain level, decreasing it beyond that point worsens liver damage [37,38]. Our findings suggest that the expression level of TNF-α in the liver of hepatic fibrotic rats was initially low, but FMGs restored its levels to normal, indicating a protective effect against liver disease by regulating the inflammatory response.

The metabolic pathway of taurine and hypotaurine, which is one of the enriched metabolic pathways, involves the metabolism of various substances, including cysteine, cysteine sulfinic acid, hypotaurine, and taurine. Apart from controlling the osmotic pressure of many tissues and cells, including the retina and the central nervous system, taurine and hypotaurine also exhibit anti-inflammatory and antioxidant properties [22]. Primarily, they prevent the formation of ROS and lipid peroxidation or defend the process of intracellular GSH storage replenishment during oxidative disorders, indirectly displaying antioxidant effects [22,23]. A decrease in taurine, known to be protective against liver damage, is reportedly linked to liver dysfunction [24]. Conversely, increased taurine levels have been observed in rats following CCl_4_ treatment [23,29]. This study revealed elevated serum levels of taurine in rats with liver damage. Notably, the levels of hepatic mRNA expression for CDO and CSAD, critical enzymes in taurine synthesis, exhibited a divergent trend compared to alterations in serum taurine levels. This divergence could be attributed to the obstruction of the taurine metabolic pathway, leading to the generation of significant quantities of taurine that neutralise oxidative stress.

Primary bile acids are synthesised through cholesterol oxidation via CYPs and secreted into the bile via transporters or receptors, including FXR, Bsep, and Mrp2. CYP7A1 functions as the enzyme that limits the rate of bile production, while FXR, a bile acid-activated nuclear receptor transcription factor, plays a crucial role in maintaining bile acid equilibrium. Bsep and Mrp2, located in bile duct membranes, regulate bile acid efflux. Bsep is a direct downstream gene of FXR, and Mrp2 transcription is also regulated by FXR [34,39].

The hepatic expression levels of CYP7A1, Bsep, Mrp2, and FXR were downregulated in rats with liver injury, consistent with prior research [40,41]. However, immunohistochemical results showed elevated expression of Bsep in injured liver tissue. This discrepancy may be attributed to the presence of transcriptional and post-transcriptional regulatory mechanisms in Bsep [42]. Nevertheless, administering FMGs significantly reversed these disruptions, suggesting that its potential mechanism for treating liver damage may involve correcting disorders in taurine and hypotaurine metabolism, as well as the primary biosynthesis pathway of bile acids.

## 5. Conclusions

This study demonstrates the potent hepatoprotective effect of FMGs on rats with hepatic injury, as evidenced by histopathological evaluation, biochemical index assays, and metabolomics analyses. Our findings solidify FMGs as a promising therapeutic option for liver injury, with their hepatoprotective effect intricately linked to the modulation of oxidative stress, inflammation, fibrosis, taurine and hypotaurine metabolism, and primary bile acid biosynthesis. This comprehensive study provides valuable insights into the mechanisms underlying the liver-protective effects of FMGs.

## Figures and Tables

**Table 1:** Primers for real-time PCR.

Gene	Forward Primer (5′-3′)	Reverse Primer (5′-3′)
TNF-α	AAGGACACCATGAGCACTGAAAGC	AGGAAGGAGAAGAGGCTGAGGAAC
TGF-β1	TATTGAGCACCTTGGGCACTGTTG	CCTTAACCTCTCTGGGCTTGTTTCC
IL-1β	GGACAGGATATGGAGCAACAAGTGG	TCATCTTTCAACACGCAGGACAGG
IL-6	GAAACCGCTATGAAGTTCCTCTCTG	GTATCCTCTGTGAAGTCTCCTCTCC
α-SMA	CAGGGAGTAATGGTTGGAATGGG	AGTTGGTGATGATGCCGTGTTC
Smad4	CTGTTGTGACTGTGGATGGCTATG	CTCTCAATCGCTTCTGTCCTGTG
Smad7	AAGAGGCTGTGTTGCTGTGAATC	ATTGGGTATCTGGAGTAAGGAGGAG
FXR	ACCTCGGCTCCTTCTCAGTTG	GGTGGCTGTGGTGAAGACTAATC
CYP7A1	ACAGAGGCCCATAGCATCCC	ACAAGGCAAAGCAGGAAGCA
CSAD	TCATCACGGAGAGCCTCAACAC	GGAGCCACCAGGACAGAAGAC
Mrp2	TAGCCTCATTCAGACGACCATCC	TTCAGGACTGCCGTATTCAACAATC
Bsep	GAAGCCATTGCCGACCAGATG	TGAGAGGACTGACAGCGAGAATC
CDO	GATTCCATTGGCTTACACCGAGTAG	TTTATGCCCTGTTCTCTGGTCAAAG

**Table 2:** Optimised MRM parameters for the determination of bile acids.

Bile Acids	Abbreviation	Parent Ion (*m*/*z*)	Daughter Ion (*m*/*z*)	DP (Volts)	CE (Volts)
α-Muricholic Acid	α-MAC	407.4	371.2	−50	−46
Cholic acid	CA	407.4	343.1	−60	−47
Chenodeoxycholic acid	CDCA	391.3	391.3	−160	−60
Deoxycholic acid	DCA	391.4	345.1	−60	−44
Glycocholic acid	GCA	464.4	464.4	−50	−8
Glydeoxycholic acid	GDCA	448.4	448.4	−150	−8
Taurocholic acid	TCA	514.4	79.9	−270	−120
Taurochenodeoxycholic acid	TCDCA	498.4	123.9	−43	−66
Tauroursodeoxycholic acid	TUDCA	498.4	79.8	−80	−120
Tauro-α-muricholic acid	T-α-MAC	514.4	514.4	−41	−15

**Table 3:** Retention time, calibration curves, linear range, and limits of quantitation of ten bile acids in standard solution.

Bile Acids	Retention Time (min)	Calibration Curves	Correlation Coefficient (r^2^)	Linear Range (ng/mL)
α-MAC	3.25	y = 6.090 × 10^−3^x + 6.530 × 10^−3^	0.9925	1–1000
CA	4.09	y = 2.123 × 10^−2^x + 4.413 × 10^−2^	0.9946	1–1000
CDCA	5.57	y = 0.2421x + 2.572	0.9945	1–1000
DCA	5.78	y = 3.328 × 10^−2^x + 3.209 × 10^−2^	0.9926	1–1000
GCA	3.13	y = 0.1203x + 0.1686	0.9951	1–1000
GDCA	4.22	y = 0.1828x + 0.3437	0.9907	1–1000
TCA	2.57	y = 2.894 × 10^−2^x + 3.643 × 10^−2^	0.9910	1–1000
TCDCA	3.36	y = 1.461 × 10^−2^x + 1.598 × 10^−2^	0.9905	1–1000
TUDCA	2.51	y = 9.325 × 10^−2^x + 1.062 × 10^−2^	0.9922	1–1000
T-α-MAC	1.95	y = 0.1631x + 0.1663	0.9908	1–1000

**Table 4:** Serum bile acid profile in three groups (n = 6, mean ± SD).

Bile Acids	Control (ng/mL)	Model (ng/mL)	FMG (ng/mL)
α-MAC	836.4 ± 398.7	3686 ± 306.3 ^###^	33.19 ± 22.42 ***
CA	3700 ± 1877	22,973 ± 2894 ^###^	368.1 ± 138.3 ***
CDCA	1110 ± 636.3	2365 ± 34.49	33.20 ± 14.90
DCA	389.6 ± 187.5	229.9 ± 57.5	54.02 ± 20.2
GCA	1411 ± 659.6	6669 ± 1532 ^##^	983.3 ± 446.4 **
GDCA	250.2 ± 125.1	908.3 ± 350.1 ^##^	223.5 ± 24.2 *
TCA	3155 ± 841.2	26,283 ± 1365 ^###^	1397 ± 561.5 ***
TCDCA	330.2 ± 159.8	1499 ± 272.6 ^###^	131.4 ± 159.8 **
TUDCA	174.8 ± 79.0	200.9 ± 57.1	131.4 ± 18.0
T-α-MAC	806.4 ± 386.4	3696 ± 1216 ^##^	643.0 ± 198.9 *

### *p* < 0.001, ## *p* < 0.01 vs. Control group; *** *p* < 0.001, ** *p* < 0.01, * *p* < 0.05 vs. Model group.

## Data Availability

The raw data supporting the conclusions of this article will be made available by the authors on request.

## Supplementary Materials

The following supporting information can be downloaded at https://www.mdpi.com/article/10.3390/metabo14080446/s1, Figure S1: Chromatograms of methanolic extract of fufang muji granules in ESI+ (A) ESI- (B) mode and 260 nm (C); Figure S2: HPLC chromatograms of mixed standards and sample of Fufang Muji granules (A-mixed standards (λ = 254 nm; B-sample of Fufang Muji granules (λ = 254 nm; C-mixed standards (λ = 325 nm; D-sample of Fufang Muji granules (λ = 325 nm) 1-galic acid; 2-vanilic acid; 3-syringic acid; 4-chlorogenic acid; 5-coffeic acid; 6-p-coumaric acid]; Figure S3: HPLC chromatograms of mixed standards and sample of Fufang Muji granules (A-mixed standards; B-sample of Fufang Muji granules; 1-cytosine; 2-quercitrin; 3-matrine; 4-sophocarpine); Figure S4: The overview of PLS-DA mode l (A, positive; B, negative) and permutation test (C, positive; D, negative); Figure S5: Abundance of differential metabolites in the three groups; Table S1: HPLC elution conditions; Table S2 Chemical composition analysis of Fufang muji granules; Table S3: Regression equations and liner rangers of six constituents (n = 6); Table S4: The contents of ten constituents in 8 batches of samples (n = 2) μg·g^−1^; Table S5: The mass fraction of polysaccharides in compound muji granules from different batches; Table S6: Summary of the differential metabolites identified in the serum of three Groups.
