# Supplementary material for: Fufang Muji Granules Ameliorate Liver Fibrosis by Reducing Oxidative Stress and Inflammation, Inhibiting Apoptosis, and Modulating Overall Metabolism
Source: Metabolites. 2024 Aug 11;14(8):446. doi: 10.3390/metabo14080446 (PMC11356414; doi:10.3390/metabo14080446)
Supplement: Supplementary file 1 [file metabolites-14-00446-s001.zip › Figure S2.pdf]

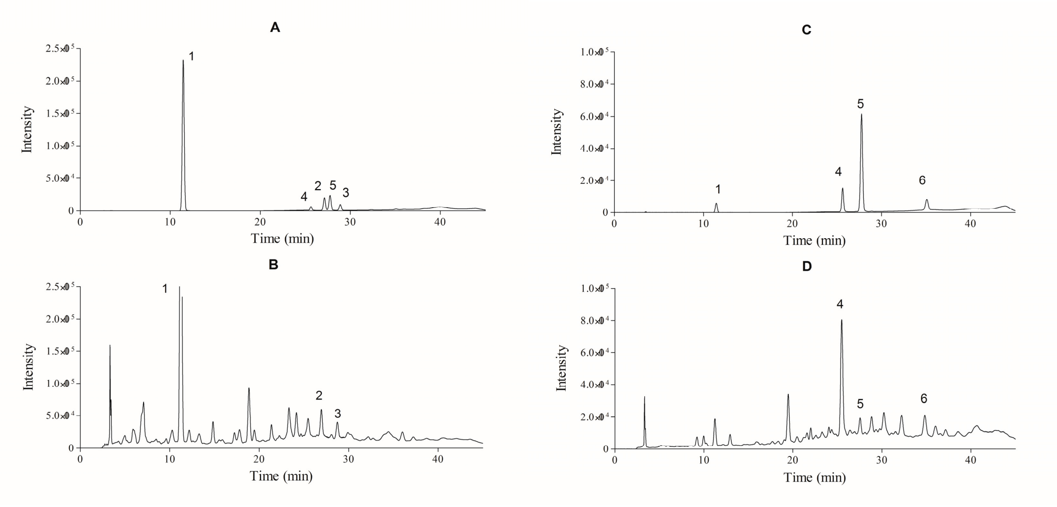

Figure S2. HPLC chromatograms of mixed standards and sample of Fufang Muji granules (A- mixed standards（λ=254nm）; B-sample of Fufang Muji granules（λ=254nm）; C- mixed standards（λ=325nm）; D-sample of Fufang Muji granules（λ=325nm） 1 - galic acid; 2-vanilic acid; 3-syringic acid;4- chlorogenic acid; 5-coffeic acid;6- p-coumaric acid]
