# Supplementary material for: Fufang Muji Granules Ameliorate Liver Fibrosis by Reducing Oxidative Stress and Inflammation, Inhibiting Apoptosis, and Modulating Overall Metabolism
Source: Metabolites. 2024 Aug 11;14(8):446. doi: 10.3390/metabo14080446 (PMC11356414; doi:10.3390/metabo14080446)
Supplement: Supplementary file 1 [file metabolites-14-00446-s001.zip › Figure S3.pdf]

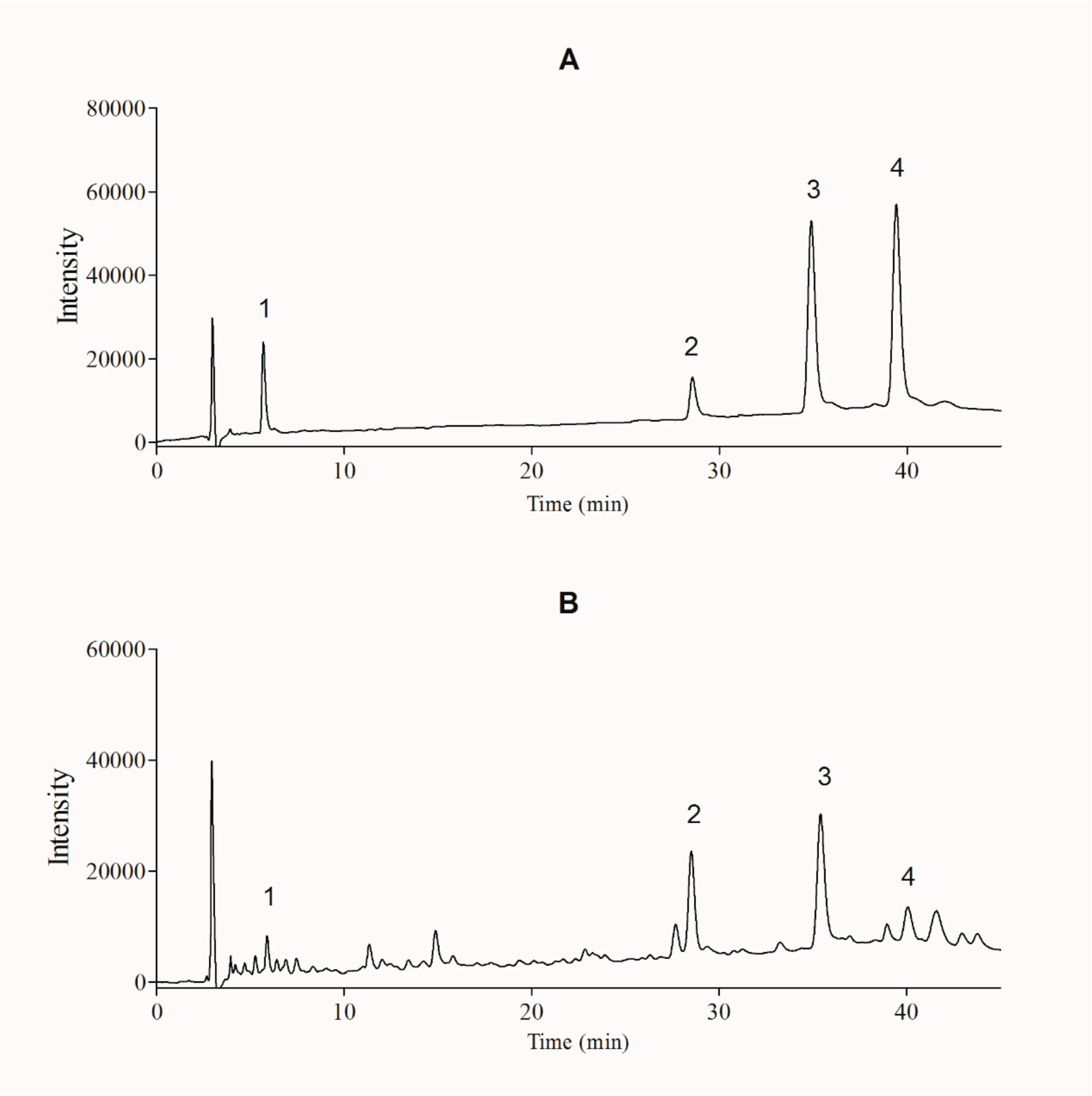

Figure S3 HPLC chromatograms of mixed standards and sample of Fufang Muji granules (A- mixed standards; B-sample of Fufang Muji granules; 1 - cytosine; 2-quercitrin; 3- matrine; 4-sophocarpine)
