# Supplementary material for: Fufang Muji Granules Ameliorate Liver Fibrosis by Reducing Oxidative Stress and Inflammation, Inhibiting Apoptosis, and Modulating Overall Metabolism
Source: Metabolites. 2024 Aug 11;14(8):446. doi: 10.3390/metabo14080446 (PMC11356414; doi:10.3390/metabo14080446)
Supplement: Supplementary file 1 [file metabolites-14-00446-s001.zip › Table S1.pdf]

Table S1 HPLC elution conditions

Time(min)	Mobile phase A(%), acetonitrile : water (0.1% formic acid), 5:95	Mobile phase B (%), acetonitrile: isopropanol: water(0.1% formic acid), 47.5:47.5:5	Flow rate (mL/min)
0	100	0	0.4
3.5	75.5	24.5	0.4
5	35	65	0.4
5.5	0	100	0.4
7.4	0	100	0.6
7.6	48.5	51.5	0.6
7.8	100	0	0.5
9	100	0	0.4
10	100	0	0.4
