# Supplementary material for: Fufang Muji Granules Ameliorate Liver Fibrosis by Reducing Oxidative Stress and Inflammation, Inhibiting Apoptosis, and Modulating Overall Metabolism
Source: Metabolites. 2024 Aug 11;14(8):446. doi: 10.3390/metabo14080446 (PMC11356414; doi:10.3390/metabo14080446)
Supplement: Supplementary file 1 [file metabolites-14-00446-s001.zip › Table S2.pdf]

Table S2 Chemical composition analysis of Fufang muji granules

No.	t_R/min	Compound	Ion mode	Detected mass	Caculated mass	Error (ppm)	Molecular formula	Fragmentation ions	λ_{max}（nm）	Type
1	8.3	Cytisine	+	191.118	191.1179	0.5	$C_{11}H_{14}N_2O$	148	218	生物碱
2	9.05	Hydroxylated sophorine	+	263.1752	263.1754	-0.8	$C_{15}H_{22}N_2O_2$	243	260	生物碱
3	10.01	Hydroxylated matrine	+	265.1913	265.1911	0.8	$C_{15}H_{24}N_2O_2$	219, 148, 205, 247, 179, 150, 164	210	生物碱
4	11.18	Hydroxylated matrine	+	265.1911	265.1911	0	$C_{15}H_{24}N_2O_2$	247, 148, 150, 205, 112, 176	210	生物碱
5	14.42	5α，9α-Dihydroxy matrine	+	281.1859	281.1860	-0.4	$C_{15}H_{24}N_2O_3$	265, 263, 148	210	生物碱
6	16.45	matrine	+	249.1963	249.1961	0.8	$C_{15}H_{24}N_2O$	148, 250, 150, 176	210	生物碱
7	17.26	Gallic acid	-	169.0141	169.0131	5.9	$C_7H_6O_5$	125	220, 270	酚酸
8	19.81	sophorine	+	247.1806	247.1805	0.4	$C_{15}H_{22}N_2O$	205, 179, 150, 148, 227, 186, 136	271	生物碱
9	20.91	14α-acetyl-matrine	+	307.2023	307.2016	2.7	$C_{17}H_{26}N_2O_3$	247, 148	274	生物碱
10	21.47	14β-acetyl-matrine	+	307.2018	307.2016	0.7	$C_{17}H_{26}N_2O_3$	247, 148	280	生物碱
11	29.97	7, 11- Dehydro-matrine	+	247.1806	247.1805	0.4	$C_{15}H_{22}N_2O$	205, 179, 150, 148，227, 136, 186，	220	生物碱
12	32.97	14α-Hydroxy-matrine	+	265.1912	265.1911	0.4	$C_{15}H_{24}N_2O_2$	205, 152, 150, 247, 229	210	生物碱

13	35.12	Sophoranol	+	265.1913	265.1911	0.8	$C_{15}H_{24}N_2O_2$	205, 152, 150, 247, 229	210	生物碱
14	44.75	4-Hydroxy-2, 6-dimethoxyphenol-1-O-β-D-[6′-O-(3″, 4″, 5″-trihydroxybenzoyl)]glucopyranoside	-	483.1135	483.11332	0.4	$C_{21}H_{24}O_{13}$	423, 271, 169	207	其他
15	44.97	chlorogenic acid	-	353.087	353.0867	0.6	$C_{16}H_{18}O_9$	191	210	酚酸
16	45.8	Quercetin-3-O-β-D-galactopyra-noside-7-O-β-D-glucopyranoside	-	625.1384	625.1399	-2.4	$C_{27}H_{30}O_{17}$	463, 301	330	黄酮
17	51.81	Vanillic acid -4-O-β-D-[6′-O-(3″, 4″, 5″-trihydroxybenzoyl)] pyranoside	-	481.0978	481.09767	0.3	$C_{21}H_{22}O_{13}$	437, 313, 271, 169	209	其他
18	52.28	syringic acid	-	197.0452	197.04445	3.8	$C_9H_{10}O_5$	182, 167, 121, 153, 138	207	酚酸
19	52.42	caffeic acid	-	179.0347	179.03389	4.5	$C_9H_8O_4$	135	271	酚酸
20	52.98	Kushenol O	+	563.1762	563.1759	0.5	$C_{27}H_{30}O_{13}$	417, 399, 297	260	黄酮
21	53.13	Bayin	+	417.1181	417.1181	0	$C_{21}H_{20}O_9$	399, 381, 351, 297, 363, 321	206	黄酮
22	54.18	1, 4, 8-Trihydroxynaphthalene-1-O-β-D-glucopyranoside	-	337.0924	337.0918	1.8	$C_{16}H_{18}O_8$	175, 301	210	萘醌

23	57.08	1, 4, 8-Trihydroxynaphthalene-1-O-β-D-glucopyranoside-(1→6)-β-D-xylopyranoside	-	469.1341	469.13405	0.1	$C_{21}H_{26}O_{12}$	175	293, 251	萘醌
24	64.43	p-coumaric acid	-	163.04	163.039	6.1	$C_9H_8O_3$	119	313	酚酸
25	65.94	Rutin	-	609.1449	609.145	-0.2	$C_{27}H_{30}O_{16}$	301, 297, 343	320	黄酮
26	66.62	Myricitrin	+	465.1037	465.1028	1.9	$C_{21}H_{20}O_{12}$	319	260, 312	黄酮
27	67.43	Hyperoside	+	465.1029	465.1028	0.2	$C_{21}H_{20}O_{12}$	303	211	黄酮
			-	463.0873	463.0871	0.4	$C_{21}H_{20}O_{12}$	403	210	黄酮
28	68.16	ellagic acid	-	300.998	300.99789	0.4	$C_{14}H_6O_8$	284, 257, 229, 185	255	酚酸
29	74.87	quercetin	-	447.0925	447.0922	0.7	$C_{21}H_{20}O_{11}$	301	212	黄酮
30	74.12	Kaempferol-3-O-glucoside	+	449.1082	449.1078	0.9	$C_{21}H_{20}O_{11}$	303	250	黄酮
31	78.15	1, 8, 9, 10- Tetrahydroxy-6H-naphthalen-[1, 2-b]-phenyl[d]-pyran-6-one-12-O-beta-D-glucoside	+	489.1028	489.1028	0	$C_{23}H_{20}O_{12}$	327	263（sharp）, 317	萘醌
32	81.45	alfonsin	-	431.0975	431.0973	0.5	$C_{21}H_{20}O_{10}$	285, 255, 243	205	黄酮
33	89.56	(7,3'-Dihrdroxy-5'-methoxyisoflavone)	+	285.0758	285.0757	0.4	$C_{16}H_{12}O_5$	123, 175, 151, 243, 270, 225, 255, 263	210	黄酮
34	97.13	naringenin	-	271.0605	271.0601	1.5	$C_{15}H_{12}O_5$	151, 177	226（sharp）	黄酮

		naringenin	+	273.076	273.0757	1.1	$C_{15}H_{12}O_5$	255, 237, 231, 249, 273	220	黄酮
35	101.05	arnebiacein	-	267.0656	267.0652	1.5	$C_{16}H_{12}O_4$	252	232（sharp）	黄酮
36	103.4	Calycosin	+	285.0759	285.0757	0.7	$C_{16}H_{12}O_5$	127		黄酮
