# Supplementary material for: Fufang Muji Granules Ameliorate Liver Fibrosis by Reducing Oxidative Stress and Inflammation, Inhibiting Apoptosis, and Modulating Overall Metabolism
Source: Metabolites. 2024 Aug 11;14(8):446. doi: 10.3390/metabo14080446 (PMC11356414; doi:10.3390/metabo14080446)
Supplement: Supplementary file 1 [file metabolites-14-00446-s001.zip › Table S3 .pdf]

Table S3 Regression equations and liner rangers of six constituents (n = 6)

sample No.	galic acid	vanilic acid	syringic acid	chlorogenic acid	coffeic acid	p-coumaric acid
170101	0.7850	0.3517	0.4867	0.7750	0.0028	0.0058
170201	0.5913	0.3300	0.424	0.6820	0.0025	0.0052
170202	0.7613	0.3200	0.4438	0.8413	0.0025	0.0075
170203	0.7388	0.3400	0.504	0.8685	0.0025	0.0075
170303	0.9296	0.4113	0.6154	0.7075	0.0027	0.0066
170304	0.7950	0.3250	0.4313	0.6613	0.0025	0.0075
