# Supplementary material for: Fufang Muji Granules Ameliorate Liver Fibrosis by Reducing Oxidative Stress and Inflammation, Inhibiting Apoptosis, and Modulating Overall Metabolism
Source: Metabolites. 2024 Aug 11;14(8):446. doi: 10.3390/metabo14080446 (PMC11356414; doi:10.3390/metabo14080446)
Supplement: Supplementary file 1 [file metabolites-14-00446-s001.zip › Table S4 .pdf]

Table S4 The contents of ten constituents in 8 batches of samples(n = 2, μg·g^{-1})

No.	cytosine	quercitrin	matrine	sophocarpine
160601	16.79	25.97	229.2	25.00
161104	12.89	27.02	210.0	25.10
170101	14.84	21.63	260.8	20.00
170201	13.58	21.16	181.5	26.32
170202	11.67	25.01	226.6	30.23
170203	14.22	30.91	211.2	26.59
170204	13.10	20.82	164.3	25.45
170301	16.08	21.21	188.9	27.31
170302	11.25	28.53	157.5	23.23
170303	15.03	21.40	226.1	27.39
170304	12.23	21.20	212.6	26.74
