# Supplementary material for: Fufang Muji Granules Ameliorate Liver Fibrosis by Reducing Oxidative Stress and Inflammation, Inhibiting Apoptosis, and Modulating Overall Metabolism
Source: Metabolites. 2024 Aug 11;14(8):446. doi: 10.3390/metabo14080446 (PMC11356414; doi:10.3390/metabo14080446)
Supplement: Supplementary file 1 [file metabolites-14-00446-s001.zip › Table S5 .pdf]

Table S5 The mass fraction of polysaccharides in compound muji granules from different batches

Batch No.	polysaccharide content(%)	Mean (%)	RSD (%)
160601	84.5	82.4	4.2
161104	77.7		
170101	84.5		
170201	78.1		
170202	78.2		
170203	87.2		
170204	86.3		
