# Supplementary material for: Fufang Muji Granules Ameliorate Liver Fibrosis by Reducing Oxidative Stress and Inflammation, Inhibiting Apoptosis, and Modulating Overall Metabolism
Source: Metabolites. 2024 Aug 11;14(8):446. doi: 10.3390/metabo14080446 (PMC11356414; doi:10.3390/metabo14080446)
Supplement: Supplementary file 1 [file metabolites-14-00446-s001.zip › Table S6.pdf]

Table S6 Summary of the differential metabolites identified in the serum of three Groups.

No.	ID	Metabolite	Adducts	Formula	Retention time (time)	m/z	Type	HMDB Subclass
1	pos	1-arachidonoyl-2-hydroxy-sn-glycero-3-phosphate	M+H, M+2Na-H, M+Na, M+H-H2O	C23H39O7P	8.4209	459.2494		-
2	pos	25-Hydroxyvitamin D3-26,23-lactone	M+H-H2O, M+H, M+H-2H2O	C27H40O4	6.7	429.2988		Vitamin D and derivatives
3	pos	DIOSGENIN	M+H-H2O, M+H, M+H-2H2O	C27H42O3	7.5605	397.3092		-
4	pos	Janthitrem C	M+H, M+Na, M+K	C37H47NO4	8.0342	570.3545		_
5	pos	Oryzalexin E	M+H-H2O, M+H, M+H-2H2O	C20H32O2	8.4209	287.236		Hydrophenanthrenes
6	pos	(E,E)-11,13-Octadecadien-9-ynoic acid	M+H-2H2O, M+Na	C18H28O2	6.5451	241.1944		Fatty acids and conjugates
7	pos	2-Phenylethyl octanoate	M+H-2H2O, M+H	C16H24O2	6.5451	213.1632		Fatty acid esters
8	pos	7,9-Illudadiene-3,14-diol	M+H-2H2O,	C15H22O2	6.5451	199.1477		Sesquiterpenoids

			M+H				
9	pos	Chrysin 7-glucuronide	M+H, M+Na	C21H18O10	2.7158	431.0965	-
10	pos	N-METHYLANTHRANILIC ACID	M+H	C8H9NO2	1.2865	152.0703	Benzoic acids and derivatives
11	pos	1-Pentanol	2M+ACN+H	C5H12O	3.7749	218.211	Alcohols and polyols
12	pos	Polyoxyethylene 40 monostearate	M+NH4	C20H40O3	6.4591	346.3306	Fatty acid esters
13	pos	N,N-dimethyl-Safingol	M+H	C20H43NO2	7.0996	330.3357	-
14	pos	Eicosanoyl-EA	M+H	C22H45NO2	7.2317	356.3512	-
15	pos	Jasmolone	M+H-2H2O	C11H16O2	6.5451	145.1009	Alcohols and polyols
16	pos	MG(19:0/0:0/0:0)	M+NH4	C22H44O4	6.4369	390.3567	Monoradylglycerols
17	pos	Tavulin	M+H-H2O	C15H20O4	5.0906	247.1322	Terpene lactones
18	pos	Wogonin	M+H	C16H12O5	4.154	285.075	-
19	pos	Acetylbalchanolide	M+NH4	C17H24O4	4.1097	310.2004	Terpene lactones
20	neg	Cholic Acid	M-H, M+FA-H, M+Cl, 2M-H	C24H40O5	6.5483	407.2804	Bile acids, alcohols and derivatives
21	neg	Sorbitan laurate	M-H, M+Na-2H	C18H34O6	5.2379	345.2283	Fatty acid esters
22	neg	N-[(3a,5b,7b)-7-hydroxy-24-oxo-3-(sulfooxy)cholan-24-yl]-Glycine	M-H, M+Na-2H	C26H43NO8S	5.6196	528.2639	Bile acids, alcohols and derivatives
23	neg	Polyporusterone A	M-H, M+Na-2H	C28H46O6	7.5532	499.3051	Bile acids, alcohols and derivatives

24	neg	3,4,5-trihydroxy-6-{[2-(2-hydroxyphenyl)-4-oxo-4H-chromen-3-yl]oxy}oxane-2-carboxylic acid	M-H	C21H18O10	2.7066	429.0829	Flavonoid glycosides
25	neg	N-Acetyl-D-phenylalanine	M-H	C11H13NO3	3.2332	206.0815	-
26	neg	[3-(7-hydroxy-4-oxo-4H-chromen-2-yl)phenyl]oxidanesulfonic acid	M-H	C15H10O7S	3.3318	333.0075	Flavones
27	neg	5b-Cyprinol sulfate	M-H	C27H48O8S	5.533	531.2999	Bile acids, alcohols and derivatives
28	neg	Coprocholic acid	M-H	C27H46O5	7.3213	449.3277	Bile acids, alcohols and derivatives
29	neg	DL-2-hydroxy stearic acid	M-H	C18H36O3	9.1734	299.2591	-
30	neg	3beta,7alpha-Dihydroxy-5-cholestenoate	M-H	C27H44O4	7.5532	431.3172	Bile acids, alcohols and derivatives
31	neg	TetraHCA	M-H	C27H46O6	6.6461	465.3224	Bile acids, alcohols and derivatives
32	neg	5-(14-Nonadecenyl)-1,3-benzenediol	M+Cl	C25H42O2	6.5483	409.2864	Benzenediols
33	neg	7-Ketodeoxycholic Acid	M-H	C24H38O5	6.1869	405.2648	Bile acids, alcohols and derivatives
34	neg	O-O-glucuronide rosiglitazone	M+Na-2H	C24H27N3O10S	5.8622	570.119	Carbohydrates and carbohydrate conjugates
35	neg	MG(0:0/20:3(11Z,14Z,17Z)/0:0)	M+FA-H	C23H40O4	5.8622	425.2912	_
36	neg	Frangulanine	M-H2O-H	C28H44N4O4	5.442	481.3175	Amino acids,

							peptides, and analogues
37	neg	Hydroxyprolyl-Isoleucine	2M+Hac-H	C11H20N2O4	4.7152	547.2945	Amino acids, peptides, and analogues
38	neg	L-(-)-Tyrosine	M-H	C9H11NO3	1.0158	180.0657	-
39	neg	Alpha-Muricholic Acid	M-H, M+Cl	C24H40O5	5.8622	407.2804	Bile acids, alcohols and derivatives
