# Supplementary figures and images for: Fufang Muji Granules Ameliorate Liver Fibrosis by Reducing Oxidative Stress and Inflammation, Inhibiting Apoptosis, and Modulating Overall Metabolism
Source: Metabolites. 2024 Aug 11;14(8):446. doi: 10.3390/metabo14080446 (PMC11356414; doi:10.3390/metabo14080446)

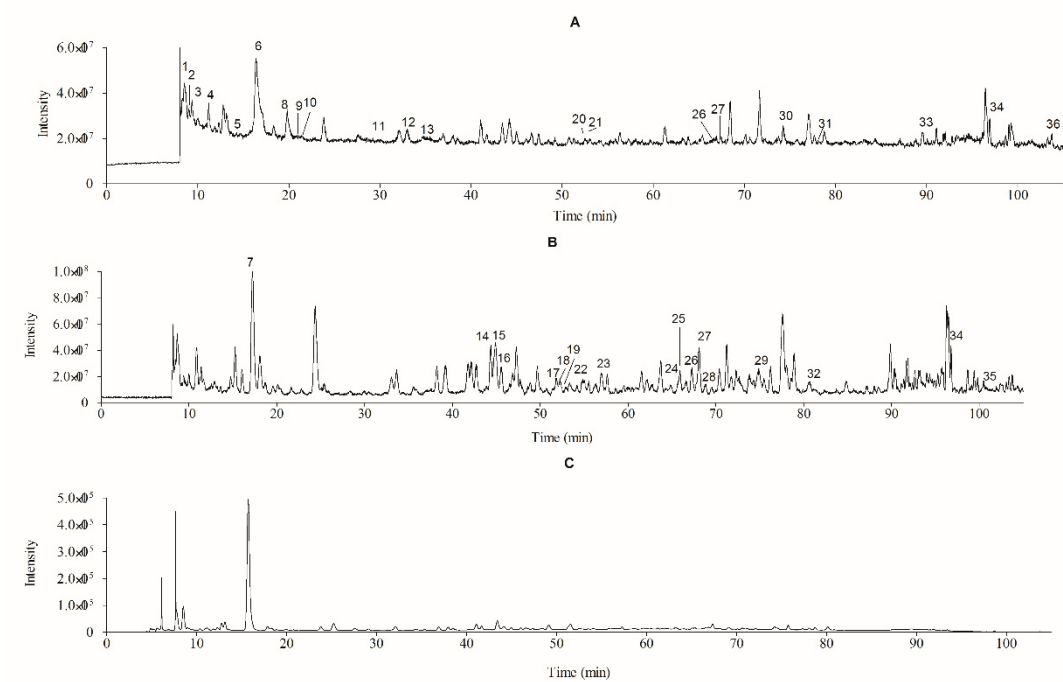

**Figure S1. Chromatograms of methanolic extract of fufang muji granules in ESI+(A)ESI- (B) mode and 260 nm(C)**

Supplement: Supplementary file 1 [file metabolites-14-00446-s001.zip › Figure S1.pdf]

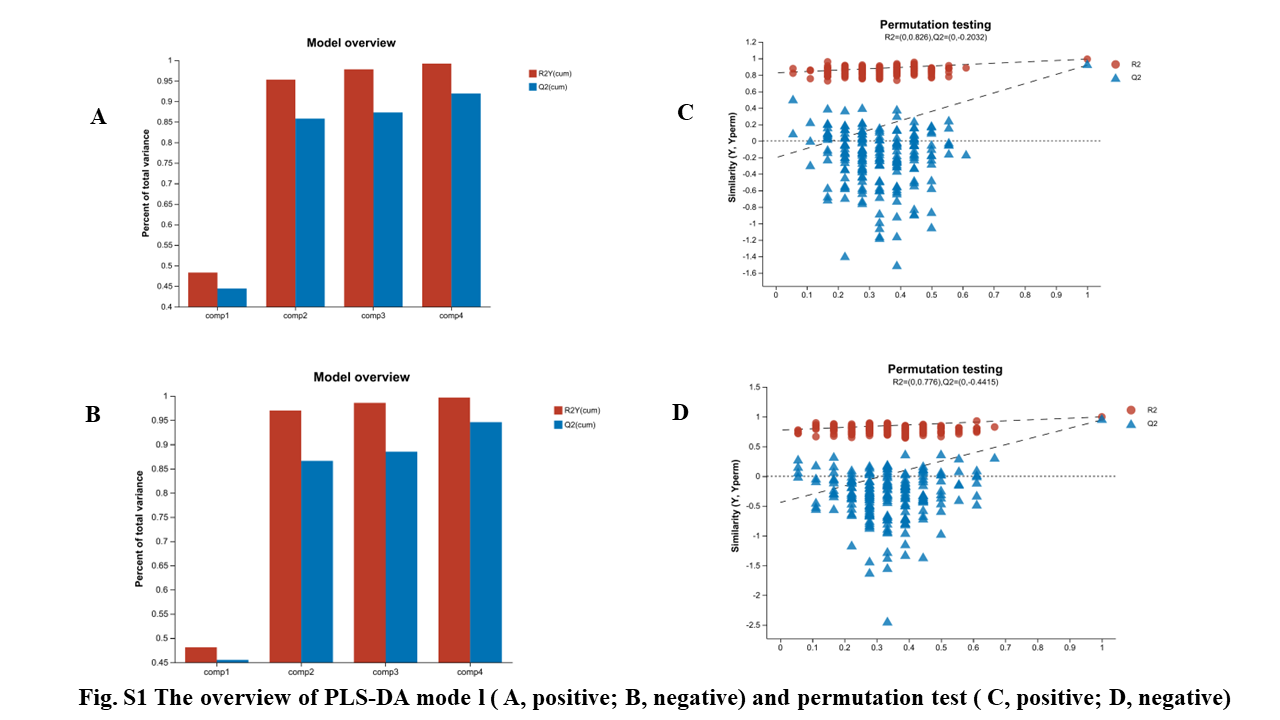

Supplement: Supplementary file 1 [file metabolites-14-00446-s001.zip › Figure S4.png]

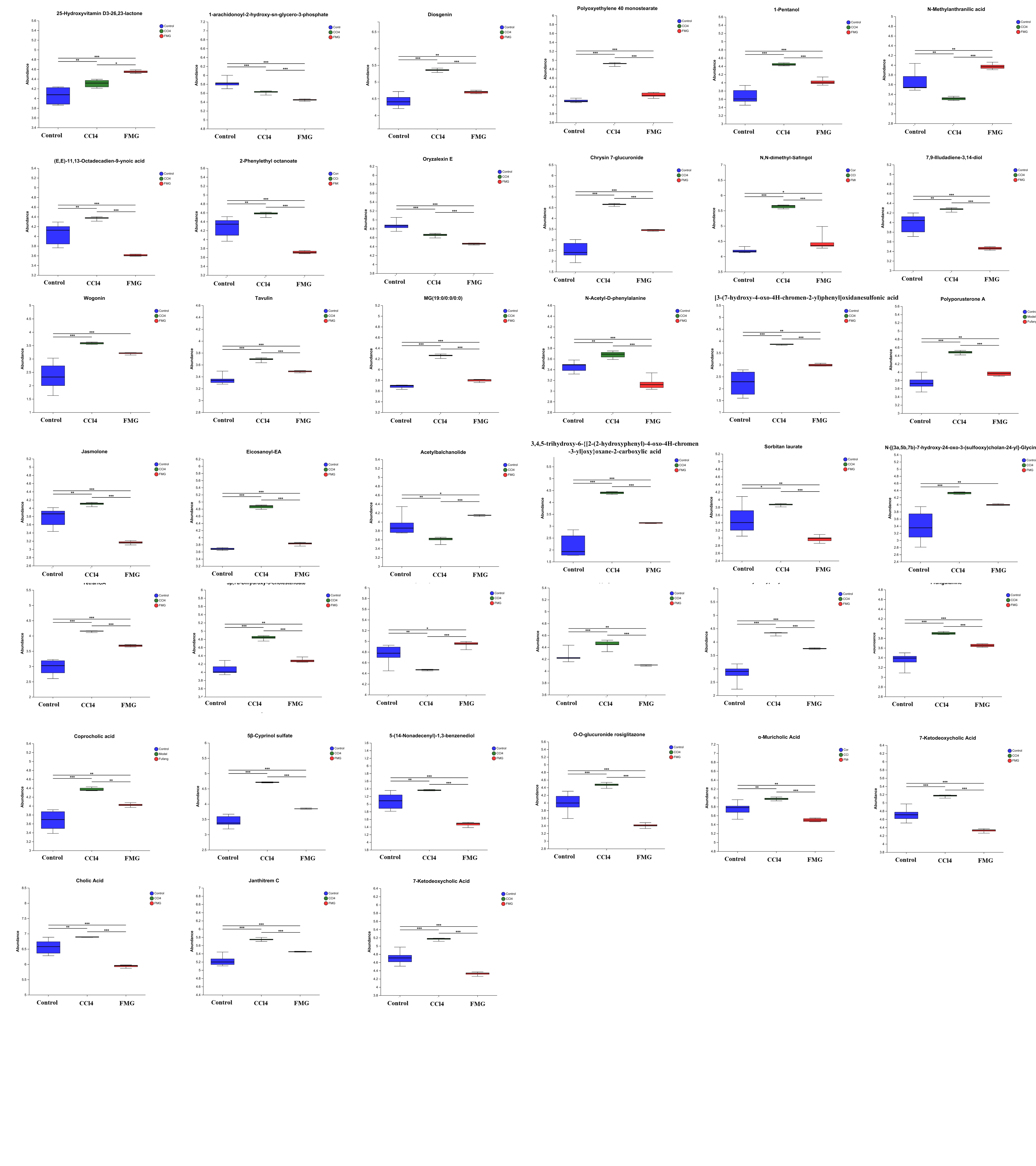

Supplement: Supplementary file 1 [file metabolites-14-00446-s001.zip › Figure S5.jpg]
